# RNA-based amplicon sequencing is ineffective in measuring metabolic activity in environmental microbial communities

**DOI:** 10.1186/s40168-022-01449-y

**Published:** 2023-06-13

**Authors:** Ya Wang, Kelsey N. Thompson, Yan Yan, Meghan I. Short, Yancong Zhang, Eric A. Franzosa, Jiaxian Shen, Erica M. Hartmann, Curtis Huttenhower

**Affiliations:** 1 Department of Biostatistics, Harvard T.H. Chan School of Public Health, Harvard University, 665 Huntington Avenue, Boston, MA 02115 USA; 2 Broad Institute of MIT and Harvard, 415 Main Street, Cambridge, MA 02142 USA; 3 Harvard T.H. Chan School of Public Health Microbiome Analysis Core, Building SPH1, 655 Huntington Avenue, Boston, MA 02115 USA; 4 Department of Civil and Environmental Engineering, Northwestern University, 2145 Sheridan Road, Evanston, IL 60208 USA; 5 Department of Immunology and Infectious Diseases, Harvard T.H. Chan School of Public Health, Harvard University, 665 Huntington Avenue, Boston, MA 02115 USA

**Keywords:** Microbial community viability, 16S rRNA transcript-based amplicon sequencing, Built environment communities

## Abstract

**Background:**

Characterization of microbial activity is essential to the understanding of the basic biology of microbial communities, as the function of a microbiome is defined by its biochemically active (“viable”) community members. Current sequence-based technologies can rarely differentiate microbial activity, due to their inability to distinguish live and dead sourced DNA. As a result, our understanding of microbial community structures and the potential mechanisms of transmission between humans and our surrounding environments remains incomplete. As a potential solution, 16S rRNA transcript-based amplicon sequencing (16S-RNA-seq) has been proposed as a reliable methodology to characterize the active components of a microbiome, but its efficacy has not been evaluated systematically. Here, we present our work to benchmark RNA-based amplicon sequencing for activity assessment in synthetic and environmentally sourced microbial communities.

**Results:**

In synthetic mixtures of living and heat-killed *Escherichia coli* and *Streptococcus sanguinis*, 16S-RNA-seq successfully reconstructed the active compositions of the communities. However, in the realistic environmental samples, no significant compositional differences were observed in RNA (“actively transcribed — active”) vs. DNA (“whole” communities) spiked with *E. coli* controls, suggesting that this methodology is not appropriate for activity assessment in complex communities. The results were slightly different when validated in environmental samples of similar origins (i.e., from Boston subway systems), where samples were differentiated both by environment type as well as by library type, though compositional dissimilarities between DNA and RNA samples remained low (Bray–Curtis distance median: 0.34–0.49). To improve the interpretation of 16S-RNA-seq results, we compared our results with previous studies and found that 16S-RNA-seq suggests taxon-wise viability trends (i.e., specific taxa are universally more or less likely to be viable compared to others) in samples of similar origins.

**Conclusions:**

This study provides a comprehensive evaluation of 16S-RNA-seq for viability assessment in synthetic and complex microbial communities. The results found that while 16S-RNA-seq was able to semi-quantify microbial viability in relatively simple communities, it only suggests a taxon-dependent “relative” viability in realistic communities.

Video Abstract

**Supplementary Information:**

The online version contains supplementary material available at 10.1186/s40168-022-01449-y.

## Background

Cultivation-free metagenomic sequencing provides an unprecedented level of detail on the composition, diversity, structure, and encoded functions of microbial communities in distinct environments. However, the ability to differentiate viable (metabolically active) microbes from nonviable (inactive) community members remains a major challenge [1] — most high-throughput profiling methods do not make this distinction (i.e., they detect both active and inactive community members), and none has been well-validated for quantifying whole-community viability [1]. The inability of most commonly used DNA-based profiling methods to distinguish viable from nonviable community members thus limits our knowledge of basic microbiome function and our ability to easily survey microbe-microbe, microbe-environment, and microbe-host interactions [2]. This is particularly true in anthropogenic, biochemically unnatural settings such as the built environment (BE), where nonviable cells often outnumber their viable counterparts [3, 4]. Despite their low abundances, viable microbes can pose health risks, especially in circulated ventilation BEs such as hospitals, clean rooms, and underground transit systems, where microbe transmission from the BE to humans is highly probable [5].

Several high-throughput sequencing-based methods have been developed for community-wise viability (activity) characterization, although their accuracy and the usage in complex microbial communities remain in debate [6, 7]. These methods integrated various modifications to differentiate sequences from live microbes vs. dead ones, including the archetypal culture-based method [8], chemical treatment that depletes DNA sourced from extracellular or nonviable (“relic”) DNA [2, 9, 10], and RNA (cDNA) amplification that inherently detects transcripts from only active cells, given that RNA degrades much faster than DNA. However, despite the wide usage in various environments, these methods tend to scale poorly to complex real-world microbial communities [6, 11]. Culture-based approaches are inherently limited to only a small fraction of “culturable” microbes and thus can only be used as a complementary validation for commensals or pathogens detected in critical environments [1, 8]. The readily used chemical-based methods — propidium monoazide staining combined with sequencing (i.e., PMA-seq) — were proved inefficient in profiling the active communities in realistic environments [6]. The performance fluctuated widely across different chemical and biological conditions and was ultimately only appropriate for very simple synthetic communities. These findings underscored the need for novel approaches which accurately determine microbial community viability in real-world settings.

As alternatives to the chemical-based methods, RNA-based methods can be utilized to characterize only the active component of microbial communities, as only biochemically active microbes should be vibrantly transcribing. Additionally, RNA molecules have a shorter half-life than DNA in the cellular and extracellular compartments, which thus reflects the viable or transcriptionally active microorganisms [12]. These factors make RNA-based methods potentially more suitable for viability assessment, including shotgun metatranscriptomics, and several marker gene methods, such as 16S rRNA transcripts or mRNA from other housekeeping genes. It should be noted that “viability” is a complex term including not only cells that are in an active growing phase, since dormant cells which may or may not produce ribosomal RNA actively can also be termed viable. RNA-based methods thus more directly correlated with current biochemical activity than viability. Therefore, in this study, we use “viable” (“or “live”) primarily referring to the metabolically active state or microbes. Shotgun metatranscriptomic sequencing, although being informative in providing both taxonomic and functional insights, has been limited in viability assays due to the challenges in sample processing and results interpretations. This is especially true in BE communities due to the low yield of molecular templates, greater (and often unknown) biodiversity, and the rapid degradation of mRNA (which can also be a benefit providing “real-time” activity status) [13, 14]. For similar considerations, amplicon sequencing based on protein-coding housekeeping genes has yet to be validated and leveraged extensively. By comparison, several unusual properties of the 16S rRNA gene have made it the most common target for amplicon-based microbial community profiling, including near-universal bacterial conservation, regions of high selection (and thus conservation) for PCR-based amplification, and a sizeable number of variable nucleotides facilitating fine-grained phylogenetic differentiation [15]. Sequencing targeting the 16S rRNA transcripts has been widely used for viability assessment in various environments, including microbial communities sampled from soil [16, 17], water [11], indoor environments [18], and human bodies [19]. In these studies, 16S rRNA transcripts and genes were amplified simultaneously for parallel RNA (cDNA) and DNA sequencing (16S-RNA-seq), the assumption being that rRNA transcript abundance can serve as a proxy for “viability” or an organism’s overall metabolic activity.

However, there are atypical properties of the 16S rRNA transcript making it potentially problematic for viability assays, given that viability is a complex multifaceted term including not only cells that are actively growing but also those in dormant states that may or may not actively transcribe ribosomal RNA. The 16S rRNA gene produces large, nonprotein-coding RNA transcripts, which are structurally much more stable than most protein-coding transcripts [12]. The persistence of stable rRNA after the cessation of other cellular activity can thus violate the biological assumption that transcripts indicate viable, or recently viable/metabolically active, microbes, while all microbes (both living and dead) will be detected in DNA libraries [6, 20]. It is thus unclear whether this technique accurately profiles the viable (or more precisely here, metabolically active) microbiome of diverse chemical and biological backgrounds. Despite its application in microbial communities from diverse environments, the reliability of 16S-RNA-seq remains to be analyzed systematically.

In this study, we provide the first systematic evaluation of RNA-based amplicon sequencing for viability (activity) assessment of human-associated and BE microbial communities. We found that while 16S-RNA-seq was able to semi-quantify activity in simple mixtures of live/heat killed microbes, it only suggested a trend of overall activity of certain taxa within complex communities. This could potentially be due to the biology of the 16S rRNA genes as well as the limitations within the 16S-RNA-seq technology.

## Methods

### Preparation of synthetic microbial communities and E. coli spike-in culture

**Fig. 1:**
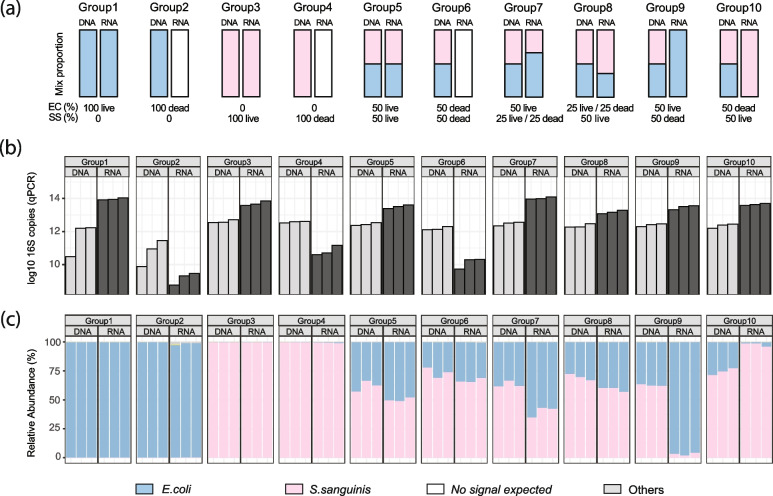
16S-RNA-seq accurately quantifies microbe viability in simple synthetic communities. **a** Expected community structures of ten live/dead *Escherichia coli* and *Streptococcus sanguinis* mixtures in DNA and RNA libraries: group (1) 100% live *E. coli* (DNA library) and 100% *E. coli* expected in RNA library; (2) 100% dead *E. coli* (DNA) and 0% *E. coli* expected in RNA libraries; (3) 100% live *S. sanguinis* (DNA) and 100% *S. sanguinis* (RNA); (4) 100% dead *S. sanguinis* (DNA) and 0% *S. sanguinis* expected (RNA); (5) 50% live *E. coli* and 50% live *S. sanguinis* (DNA), same proportion expected in RNA libraries; (6) 50% dead *E. coli* and 50% dead *S. sanguinis* (DNA), no signal expected in RNA libraries; (7) 50% live *E. coli*, 25% live *S. sanguinis*, and 25% dead *S. sanguinis* (DNA) and 67% *E. coli* and 33% *S. sanguinis* (RNA); (8) 25% live *E. coli* and 25% dead *E. coli*, 50% live *S. sanguinis* (DNA), and 67% *S. sanguinis* and 33% *E. coli* (RNA); (9) 50% live *E. coli*, 50% dead *S. sanguinis* (DNA), and 100% *E. coli* (RNA); and (10) 50% dead *E. coli* and 50% live *S. sanguinis* (DNA) and 100% *S. sanguinis* (RNA). **b** 16S rRNA gene copy numbers detected from the DNA and RNA extractions from 10 synthetic cultures. **c** Relative abundances of synthetic community members by 16S-RNA-seq in DNA and RNA libraries. Each experiment had three biological replicates. As expected, the experimental results closely follow the predicted simple community composition, albeit small fluctuations in the relative abundance of the microbes

We constructed ten synthetic microbial communities (Fig. 1a and Supplementary Table 1) comprising active or heat-killed *E. coli* strain ATCC 47,076 and *S. sanguinis* strain ATCC 10,556. The bacteria were subcultured on brain–heart infusion (BHI) agar and incubated overnight at 37 °C in room air (for *E. coli*) or 5% CO2 (for *S. sanguinis*). The bacteria were then inoculated into 5 mL fresh BHI broth and incubated at 37 °C while shaking at 250 rpm until early log-phase growth (*OD*_600_ = 0.1) was achieved. The cultures were adjusted to 10^5^ CFU/ml by serial tenfold dilution in BHI broth. For each of the strains, half of the cultures were killed by heat at 75 °C for 10 min (for *E. coli*) or 65 °C for 30 min (for *S. sanguinis*). The heat-killed bacteria were then mixed with live culture proportionally as shown in Fig. 1a to a final volume of 1 mL. The ten 1-mL cultures were then proceeded with DNA/RNA parallel extraction as described below. Each group was prepared in three replicates for statistical comparisons.

To explore the quantitative potential of 16S-RNA-seq in realistic complex communities, we spiked known amounts of *E. coli* cells into microbial samples collected from computer screens, computer mice, soil, and human saliva. As calibrated previously [6], the bacterial biomass (16S rRNA gene copies) on computer screens and computer mice was roughly equivalent to 10^2^–10^3^ *E. coli* cells, while the bacterial biomass observed from soil and saliva were estimated at 10^6^–10^7^ cells. Therefore, the low biomass samples (computer screens and computer mice) were spiked with ~ 500 *E. coli* cells (0.5 mL × 10^3^ CFU/ml), and for high biomass samples (soil and saliva), we spiked with 10^6^ *E. coli* cells (1 mL × 10^6^ CFU/ml).

### Sample collection

**Fig. 2:**
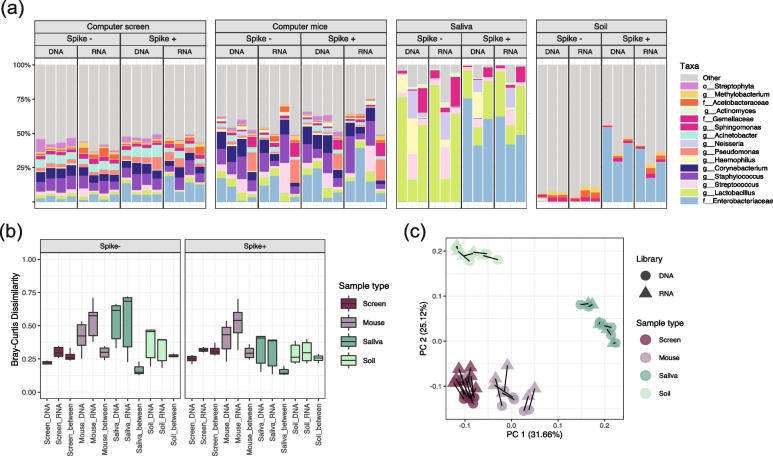
16S-RNA-seq was not able to differentiate active vs. whole microbiome in spiked realistic communities. Our second evaluation of 16S-RNA-seq used four environmental microbial community types (high and low biomass and high and low expected activity) spiked with varying levels of cultured/heat-killed *E. coli*. **a** Relative abundances of 15 taxa detected with the highest mean abundance across all samples with clear differences between sample types. Four biological replicates were taken for computer screens and mice and three biological replicates for saliva and soil. **b** Bray–Curtis dissimilarity within and between communities from DNA and RNA libraries indicated no significant differences between the RNA (cDNA) and DNA pairs (PERMANOVA *R*^2^: 2.3%, FDR *q*: 0.254). The RNA (cDNA) libraries showed the highest inter-replicate dissimilarity, followed by the DNA in most cases. Columns labeled with the “sample_DNA” (e.g., Screen_DNA) show dissimilarities within the indicated DNA libraries. Those annotated “type_RNA” (e.g., Screen_RNA) show calculations within the RNA libraries, and “type_between” (e.g., Screen_between) represents distances between paired samples in DNA vs. RNA libraries. **c** After constructing an ordination based on each sample’s pairwise Bray–Curtis dissimilarity, the dissimilarities were largely explained by sample types. With the screens and mice ordinating closest together, as expected. Lines connect identical samples in DNA and RNA libraries

Samples used in the spike-in experiment (Fig. 2) were collected on 8 July 2019, from four separate computer screens, computer mice, soil environments, and human saliva. Each target was sampled using two replicates, one to be spiked with *E. coli* culture and the other as a control. The computer screens and computer mice were sampled using two FLOQSwabs (Copan) in parallel. The swabs were pre-moisturized in autoclaved 0.85% saline. The entire surface of each computer screen and each computer mouse was swabbed for 1 min and 30 s, respectively. The saliva samples were collected as described previously [21]. Briefly, the subjects were asked to let saliva collect in their mouth for 1 min. Approximately, 1.5 mL saliva was collected into a labeled 2 mL sterile tube. The soil samples were collected from four flowerpots around the Harvard T.H. Chan School of Public Health (655 Huntington Ave., Boston, MA, USA). Two vials of samples, each containing 0.2 g of soil, were obtained from each site.

**Fig. 3:**
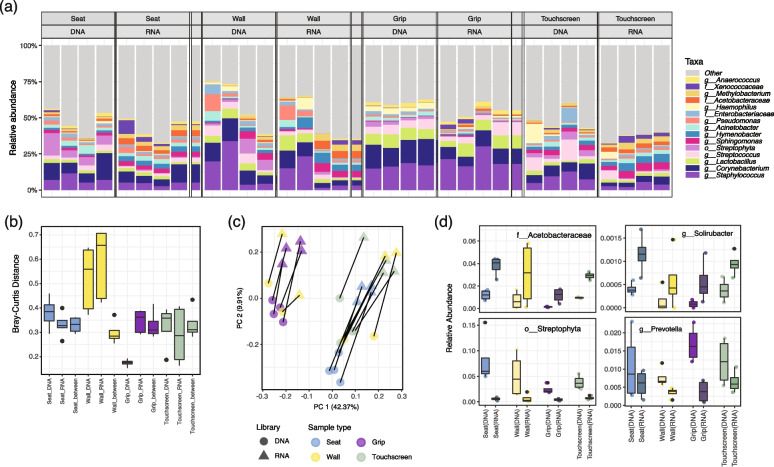
16S-RNA-seq indicated subtle differentiation between DNA and RNA libraries in samples from the Boston (MBTA) subway system. **a** Relative abundances of the 15 taxa with the highest means across four sample types in DNA and RNA libraries indicated overall similar taxonomic compositions. Each column represents a biological replicate. **b** Bray–Curtis distance distributions between MBTA samples within/between DNA and RNA libraries. **c** Principal coordinate analysis (PCoA) of MBTA samples using Bray–Curtis distances. Sample type and library type are both drivers of overall community composition. **d** Four differentially abundant taxa that consistently enriched or depleted in RNA libraries across sample types. The Acetobacteraceae family and *Solirubacter* genus were consistently enriched in RNA libraries, while the Streptophyta order and the human commensal *Prevotella* genus were under quantified in RNA libraries (mixed-effects linear models, *q*-value = 0.002, 0.003, 0.002, and 0.012, respectively)

To assess BE microbial communities (Fig. 3), we collected 16 samples from the Massachusetts Bay Transportation Authority (MBTA) Green Line E on 19 August 2019, including four samples each from the seats, walls, grips, and touchscreens of the ticket machines, as previously described [22]. The MBTA approved all aspects of our transit system sampling and gave permission to the Harvard T.H. Chan School of Public Health to conduct this study (Supplementary Fig. 1). Sampling of the seats, grips, and walls was conducted in train cars as the train proceeded from the Longwood Station towards Park Street. Station samples were collected by swabbing the entire surface of touchscreens of ticket machines for 1 min at the Park Street Station using one pre-moisturized swab.

Swab samples were also collected from pure *E. coli* culture, blank culture broth, indoor and outdoor air, extraction reagent (EB buffer), water, and 0.85% saline in the lab space as negative controls for the environment (Supplementary Fig. 2).

### DNA/RNA extraction and real-time qPCR assay

Total DNA and RNA from synthetic communities and natural microbial communities were extracted using the AllPrep PowerFecal DNA/RNA Kit (Qiagen) following the manufacturer’s instructions, with concentrations quantified using a Qubit 2.0 fluorometer (Invitrogen, Carlsbad, CA, USA). RNA samples were reverse transcribed into cDNA using QuantiTect Reverse Transcription Kit (Qiagen). Real-time quantitative polymerase chain reaction (qPCR) was performed using universal primers targeting a 466-bp region in 16S rRNA V4 region [23]. Each 20 μL reaction mixture consisted of 10 μL 2 × KAPA SYBR FAST qPCR Master Mix (KAPA Biosystems), 0.4 μL (a final concentration of 10 μM) forward and reverse primers, 8.2 μL PCR grade water and 1 μL of DNA template (for bacterial cultures, soil and saliva samples), or 4.2 μL PCR grade water and 5 μL of DNA template (for computer screen and computer mouse surfaces). The thermocycling program was as follows: (1) initial denaturation for 3 min at 95 °C, (2) 40 cycles of 3 s at 95 °C and 20 s at 60 °C, followed by (3) a melting curve in the range of 60 to 95 °C. Standard curve was generated in each batch of runs using serial dilutions of pSPIKE-P (Addgene Plasmid no. 101172) — a plasmid of known size and with 16S rRNA V4 region insertion.

### 16S rRNA amplicon sequencing

A modified protocol adapted from the Earth Microbiome Project [15] and the Human Microbiome Project [24] was applied on the synthetic community samples (part 1), spiked experiments (part 2), and the samples from the MBTA system (part 3). In brief, DNA and cDNA were subjected to 16S amplification using primers incorporating the Illumina adapters and a sample barcode, allowing directional sequencing over the 16S rRNA gene variable region V4 (16S-RNA-seq). Each 25 μL PCR reaction contained 10 μL of 2 × HotMasterMix with the HotMaster Taq DNA Polymerase and 5 μL of primer mix (2 μM of forward primer 515F and 2 μM barcoded reverse primer 806R). The thermocycling conditions comprise an initial denaturation of 94 °C for 3 min, followed by 30 cycles of denaturation at 94 °C for 45 s, annealing at 50 °C for 60 s and extension at 72 °C for 90 s, and a final extension at 72 °C for 10 min.

We followed the Illumina® (Illumina Inc., San Diego, CA, USA) 16S Metagenomic Library Preparation guidelines to create 16S rRNA amplicon libraries from keyboard and human fecal samples (part 4). Briefly, we amplified the 16S rRNA gene V4 region using primers with Illumina® sequencing adaptors and barcodes [25, 26]. Three amplicon PCR replicates were performed for each sample to control for PCR bias [27, 28]. The initial amplicon PCR was performed in 25 μL reactions using 2.5 μL (for stool) or 5 μL (for keyboard samples) of input DNA/cDNA, 1.0 μM forward and reverse primers, and 12.5 μL of KAPA Taq HiFi HotStart High Fidelity ready mix (KAPA Biosystems, Woburn, MA, USA). Cycling conditions for the amplicon PCR started as 95 °C for 3 min, followed by 25 cycles of 95 °C (30 s), 50 °C (30 s), and 72 °C (30 s), with a final extension at 72 °C for 5 min. Triplicate amplicon reactions originating from the same DNA/cDNA sample were pooled and cleaned using AMPure® XP beads (Agencourt Biosciences, Beverly, MA, USA) followed by an indexing PCR using Nextera XT Index Primer Set A (Illumina, Inc., San Diego, CA, USA). Cycling conditions for the indexing PCR were 95 °C for 3 min, followed by eight cycles of 95 °C (30 s), 55 °C (30 s), and 72 °C (30 s) with a final extension at 72 °C for 5 min. Size and quality of all the indexed libraries were checked. Libraries were quantified and pooled to a final concentration of 4 nM and then sequenced using Illumina MiSeq platform with addition of 15% PhiX and yielded paired-end reads of 150 bp in length in each direction.

### Sequencing data analysis

Taxonomic profiles were generated with the bioBakery 16S workflow [29] v0.12.1 (http://huttenhower.sph.harvard.edu/biobakeryworkflows). In brief, paired-end reads were demultiplexed using EA-Utils [30] and then filtered, dereplicated, and clustered into referenced-based operational taxonomic units (OTUs) using USEARCH v7.0.1090 [31] at 97% similarity or the default settings. Taxonomic annotations were assigned using USEARCH algorithms against the Greengenes 13_8 database [32], and quantified hits were built into an OTU feature table (Supplementary files 2 and 5). Relative abundance was calculated per sample by taking each feature and dividing it by the total read counts.

### Statistical analyses

An in-house R-script employing the libraries stringr, dplyr, matrixStats, vegan, ape, ggplot2 [33], phytools [34], scales, phyloseq [35], and GuniFrac [36] was used to compare the outputs from bioBakery workflows. First, OTUs were condensed to the genus level if possible; when a genus was not assigned, a sum abundance was calculated at each OTU’s terminal taxonomic level. The resulting clades are referred to generally as taxa. Taxa were then passed through a filter of > 0.01% relative abundance in at least 10% of all samples. Principal coordinate analysis (PCoA) was performed using Bray–Curtis dissimilarity based on these relative abundances. Univariate tests were performed using PERMANOVA with respect to sample types, library type, spike-in effect (for part 2), and studies (for part 4) (statistical results in Supplementary file 3). In brief, Bray–Curtis dissimilarity was compared within communities from DNA or RNA (cDNA) libraries (labeled as sample_DNA or sample_RNA in Fig. 2b and Fig. 3b) and in RNA (cDNA) and DNA pairs (labeled as sample_between). qPCR results on the ten synthetic communities were compared using paired *t*-test using GraphPad Prism (version 8). Multivariate tests for taxa associated with metadata (Supplementary files 1 and 4) were performed using MaAsLin2 (Microbiome Multivariable Association with Linear Models) with default settings [37]. For this analysis, we included three types of metadata as covariates: sample type, library type, spike-in effect (for part 2), and studies (for part 4).

## Results

### 16S-RNA-seq successfully reconstructs the viable fraction in simple synthetic communities

As mentioned, several properties of the 16S rRNA gene have made it favorable but also potentially problematic for activity assessment. To evaluate the degree of inherent biological and technical effects, we tested this methodology in ten synthetic communities composed of active and heat-killed *Escherichia coli* and *Streptococcus sanguinis* mixed in known proportions (Fig. 1a). The active/overall bacterial load in each of the mixed cultures was estimated by qPCR targeting the 16S rRNA V4 gene region and then profiled using sequencing (Fig. 1 b–c). Readouts from RNA (cDNA) samples would be expected to linearly correlate with the active proportions of microbes in each group, both in absolute quantification (qPCR) and in compositional profiling (sequencing) results. Per this expectation, 16S-RNA-seq accurately profiled the active fraction in groups containing a monoculture of active cells (group 1: 100% live *E. coli*; group 3: 100% live *S. sanguinis*; group 9: 50% live *E. coli*; and group 10: 50% live *S. sanguinis*, Fig. 1 b–c). In cultures containing mostly dead cells (groups 2, 4, and 6), which would ideally result in “blank” sequencing results from RNA libraries, the small number of sequences still obtained was from *E. coli* or *S. sanguinis*, likely due to low-level contamination from persistent rRNA molecules or bleed-through from other samples (as would occur in any near-empty amplicon library, regardless of activity). Most importantly, the overall RNA amounts in these groups (2, 4, and 6) were supported by much lower RNA yields and qPCR signals (paired *t*-test, *p*-value = 0.0335, 0.0079, and 0.0142 for groups 2, 4, and 6 qPCR, respectively. Supplementary files 1 and 3). In mixed culture groups (group 5: 50% live *E. coli* and 50% live *S. sanguinis*; group 7: 50% live *E. coli* and 25% live *S. sanguinis*; and group 8: 25% live *E. coli* and 50% live *S. sanguinis*), the composition of the two microorganisms agreed with the known proportions, while the abundances differed slightly. 16S rRNA gene copies were higher in RNA (cDNA) samples compared to the DNA ones as detected by qPCR, except for the groups containing mostly “dead” cells (i.e., groups 2, 4, and 6). This is concordant with the long half-life of ribosomal RNA molecules and their transcription during normal growth in culture [12]. Overall, 16S-RNA-seq was able to semiquantitatively (and at least qualitatively) differentiate viable from nonviable microbes in extremely simple synthetic “communities.”

### 16S-RNA-seq was not able to differentiate the active fraction of microbial communities in spiked realistic communities

To validate the performance of 16S-RNA-seq in more complex communities, and to further explore its quantitative potential, we applied the assay on a collection of human and environmentally sourced microbiome samples. These included two high-biomass, high-complexity communities (i.e., soils and human saliva) and two relatively low-biomass communities sourced from low-viability environments (i.e., computer screens and computer mice). Each of these sample types were spiked with a 1 mL *E. coli* culture in which live:dead cells were mixed at the ratio of 1:1. We intended to compare the absolute amount of known spike-in cultures in different panels by considering its relative abundance and the absolute quantification from qPCR (“Methods”) [6]. Similar to the mock communities, qPCR signals from RNA (cDNA) samples were higher compared to the DNA ones (Supplementary Fig. 3, paired *t*-test, *p*-values = 0.0002, 0.0038, 0.0014, and 0.0133 for computer screen, mouse, saliva, and soil, respectively), yet no apparent differences were observed between the samples with(out) spike-in cultures (paired *t*-test, *p*-values in Supplementary file 3). The addition of *E. coli* controls into the spiked-in (“spike + ” vs. “spike − ”) groups increased the relative abundance of Enterobacteriaceae in computer screens, soil and human saliva (mixed-effects linear models, *q*-values: 0.02, 4.08E-5, and 0.01 for computer screen, soil and saliva samples, respectively). However, the quantification using Enterobacteriaceae relative abundance and 16S rRNA qPCR in RNA vs. DNA samples did not reliably correlate the absolute *E. coli* amounts with the actual live:dead proportion in spike-in cultures. No consistent increase (or decrease) of Enterobacteriaceae relative abundance was observed in RNA libraries vs. DNA ones within each environment (mixed-effects linear models, *q* = 0.64, 0.99, 0.97, and 0.87 for computer screen, soil and saliva), much less cross the environments (mixed-effects linear models, *q* = 0.97). The absolute *E. coli* amounts were not significantly different in RNA vs. DNA samples (paired *t* test, *p* = 0.08, 0.14, 0.09, and 0.16 for computer screen, mouse, human saliva, and soil, respectively). These likely suggest that these environments are biologically different in terms of microbial composition and activity, while 16S-RNA-seq is not able to profile such differences, let alone quantify them.

Within these spiked community samples, 16S-RNA-seq produced almost no compositional differentiation between DNA vs. RNA libraries (Bray–Curtis dissimilarity median 0.28–0.33 within sample) (Fig. 2 a–b). The compositional differences across all samples were largely explained (*R*^2^ = 61.3%) by sample type (PERMANOVA FDR *q* = 0.0015), but not library type (*R*^2^ = 2.3%, FDR *q* = 0.254). This was also reflected in the principal coordinate analysis (PCoA) plot, where samples were grouped by types but not by libraries (Fig. 2c). These results are suggestive that 16S-RNA-seq is not able to capture the truly active fraction of the whole microbial community in complex realistic samples.

These results suggest that the assumption that 16S rRNA amplicons are directly enriched for viable, actively growing microbes in complex communities is likely incorrect or at least highly context dependent. This is potentially due to the unusual properties of 16S rRNA genes as mentioned above (especially stability), in addition to further unique behaviors such as variable copy numbers of the rRNA gene across different microbial genomes, as well as variable transcription levels in different growth phases within single microbes [38, 39]. It is thus plausible that 16S rRNA abundance thus does not universally, quantitatively correlate with underlying microbial cell count by nature. The activity quantification is likely to be especially affected by the unique stability of this nonprotein-coding RNA, which has been reported detectable for days or even months after microbes’ death [12]. This would also be influenced by context-specific effects on 16S-RNA-seq, as more diverse microbial communities (realistic BE communities vs. simple synthetic cultures) harbor more variation in terms of the genomic 16S rRNA gene copies, growth status, and chemical influences on the transcripts’ stability. There are thus several reasons why 16S rRNA amplicons may not accurately reflect microbial activity outside of very simple, synthetic communities as initially expected.

### Subtle differentiation between DNA and RNA libraries reflects RNA biochemistry rather than true activity

When 16S-RNA-seq was applied to the subway systems, distinct results were observed from those of more complex compositions (e.g., saliva and soil in “Results” part 2). Across BE sample types, the relative abundance of the more abundant taxa remained consistent in DNA and RNA libraries (Fig. 3a). Although the Bray–Curtis dissimilarities between DNA and RNA libraries remained low (median: 0.34–0.49) (Fig. 3b), a significant difference between library types was observed (PERMANOVA FDR *q* = 0.005, *R*^2^ = 0.117). However, sample type still remained as the major (*R*^2^ = 34.2%) driving effect of the compositional differences in the BE communities (FDR *q* = 0.002). This was also reflected in the ordination analysis, where samples were differentiated by sample source as well as by library type (Fig. 3c). The differentiation between RNA and DNA libraries, though subtle, indicated that the RNA-derived communities could be somewhat distinguished from the whole consortium in microbial samples of similar chemical or biological backgrounds as identified by 16S-RNA-seq; whether or not such differences were indicative of true activity quantitatively remains unelucidated.

The compositional dissimilarity in DNA vs. RNA libraries resulted from differentially abundant taxa, several of which increased (or decreased) consistently between DNA and RNA libraries across sample types despite the overall stability (Fig. 3d). Most of the taxa enriched significantly in RNA communities were typically environmentally sourced microbes that existed in relatively low abundance, such as the Acetobacteraceae family (mixed-effects linear models, *q*-value = 0.002), the *Solirubrobacter* genus (mixed-effects linear models, *q*-value = 0.003), and the *Pilimelia* genus (mixed-effects linear models, *q*-value = 0.039). Intriguingly, several highly abundant taxa commonly found in human or built environments (i.e., *Streptophyta*, *Prevotell*a, *Lactococcus*, *Corynebacterium*, and *Propionibacterium*) were found depleted in RNA libraries across different sample types (Fig. 3d and Supplementary Fig. 4, mixed-effects linear models, *q*-values in Supplementary file 6). The decreased proportion of human commensals intuitively indicates compromised activity in the built environment. However, it is possible that these decreases simply indicate relative increases of the other taxa, considering that the environmental-related taxa (such as *Streptophyta*) appeared enriched in the same way. Alternatively, this would suggest that some microbes are simply more or less visible in 16S rRNA vs. DNA amplicons due to the rRNA production rate, differences in DNA vs. cDNA amplification efficiency, rRNA molecules stability, or growth phase of the underlying microbes, regardless of their actual activity.

### 16S-RNA-seq suggests “relative activity” trends of several taxa across different sample types

**Fig. 4:**
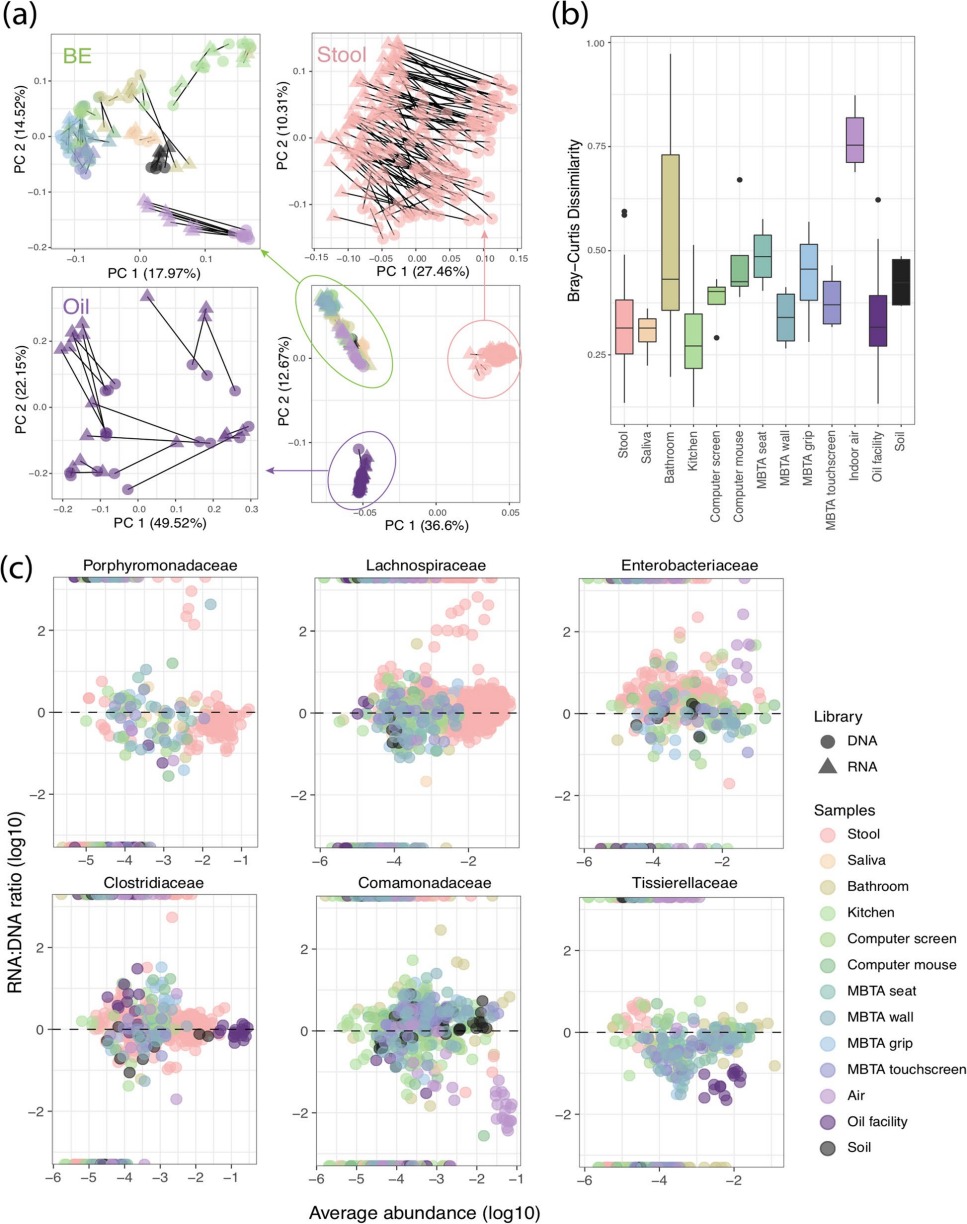
16S-RNA-seq indicated that some taxa may be more or less viable depending on the environment types. **a** PCoA analysis using Bray–Curtis dissimilarities among filtered OTUs. Sample type is the major contribution to the overall compositional dissimilarities (*R*^2^ = 64.3%, FDR *q* = 0.001), while library type also drives compositional change in samples of similar sources (*R*^2^ = 2.0%, FDR *q* = 0.001), suggesting that 16S-RNA-seq provides some differentiation between DNA vs. RNA libraries in similar samples. **b** Bray–Curtis distance distributions within/between DNA and RNA libraries. Generally, BE samples tend to have higher dissimilarity; indoor air samples differ most between DNA and RNA libraries. **c** RNA/DNA relative abundance ratios of genus in Porphyromonadaceae, Lachnospiraceae, Enterobacteriaceae, Clostridiaceae, Comamonadaceae, and Tissierellaceae. Overall trends of “relative activity” were suggested in these families by 16S-RNA-seq

Numerous factors are thought to account for compositional differences in RNA libraries in 16S-RNA-seq [12]. To assess consistency in community composition changes across different studies and sample types, we compared our results with previous 16S-RNA-seq studies from various sources (i.e., household surfaces [18], indoor air [40], oil production facilities [41], and human stool [19]). We uniformly re-profiled each study’s raw data to ensure a consistent process of taxonomic assignment and reduce bias (“Methods,” Supplementary file 5 and Supplementary Fig. 5). The samples were clearly clustered by environmental sources in principal coordinate analysis, suggesting that sample type remains the most important contribution to compositional differences (PERMANOVA FDR *q* = 0.001, *R*^2^ = 64.3%, Fig. 4a). Library type also exhibited small yet significant differences in samples from MBTA environments, indoor air, and stool samples, which agrees with above observation (Fig. 3c).

This joint ordination analysis suggested that 16S-RNA-seq somewhat differentiated viable bacteria from whole communities in samples of similar origins. Particularly, samples from indoor air showed the highest compositional dissimilarities between library types (Fig. 4b). This suggests that 16S-RNA-seq could potentially discriminate active from non-active communities in these abiotic environments where desiccation and limited nutrients impose selection. Contrarily, the dissimilarities between the RNA and DNA libraries were lower in communities with likely readily growing microbes (compared to air samples), including stool, bathroom and kitchen surfaces, computer mouses, and MBTA train walls. This indicated a potential lack of discriminatory ability of this methodology in these environments, as we would still expect relic DNA from dead microbes. These differences reiterated that 16S-RNA-seq results are likely influenced by factors such as biochemical characteristics of the samples, initial microbial compositions and growth, community diversity, and bacterial load.

The above observations suggested that 16S-RNA-seq provides significant community differentiation in some cases, but not always. We next focused on taxa-wise characteristics, to understand which microbes were consistently differentiated by 16S-RNA-seq. We identified 197 differentially abundant taxa in DNA vs. RNA libraries using mixed-effects linear models [42] (FDR *q* < 0.25) (Supplementary file 6). Six prevalent families (Porphyromonadaceae, Lachnospiraceae, Enterobacteriaceae, Clostridiaceae, Comamonadaceae, and Tissierellaceae) containing the greatest number of significantly differentiable genera were selected for further analysis of the relative abundance changes using the RNA/DNA abundance ratio between RNA and DNA libraries (“Methods”).

The RNA/DNA ratio (i.e., a measurement normalizing RNA abundance with underlying taxonomic abundance) was used as an index to infer the metabolic activity/viability potential in previous studies [1, 40]. Although the majority of samples had approximately the same relative abundance in DNA and RNA libraries, 16S-RNA-seq indicated the trend of the family’s potential activity across different sample types (Fig. 4c). For example, most genera in the Enterobacteriaceae family were more abundant in RNA libraries than in DNA libraries, suggesting that they are more likely to be active compared to Porphyromonadaceae, where most genera were depleted in RNA libraries. Notably, RNA/DNA ratios are more reliably interpreted in taxa initially abundant in either DNA or RNA libraries (e.g., the low RNA abundances in Comamonadaceae and Tissierellaceae taxa in indoor air and oil facility samples indicated their being less active), given that the relative abundances are affected both biologically and technically. The abundance changes of rare taxa are thus more likely to be exaggerated by sampling effects, insufficient sequencing depth, and underlying taxa changes (i.e. DNA abundances). This partially explains the “phantom taxa” (taxa only found in RNA libraries while not DNA libraries) [20, 43] in each family across different samples (Fig. 4c).

## Discussion

The role of rRNA as an indicator of microbial activity has long been scrutinized, with the concern that the correlation between real-time microbial activity and rRNA quantities in environmental samples is inherently inconsistent due to differences across microbes biologically and across environments [12, 44, 45]. Before this limitation could be addressed, 16S-RNA-seq became an established method for microbial community activity assessment and has been applied in various environments [11, 16–18]. The reliability of this technique remains to be tested across different applications and is especially needed in complex microbial communities. This study performed a systematic evaluation of 16S-RNA-seq using synthetic, spiked realistic communities, and environmental microbial communities and for the first time explored its quantitative potential. 16S-RNA-seq was qualitatively appropriate and provided at least semiquantitative evaluation on active taxa in simple synthetic communities. However, this approach was unable to distinguish active microbes from relic DNA in complex realistic situations, with the differentiation most likely affected by the sustainability of rRNA molecules of each taxon and underlying growth rates of the microbes rather than viability per se (e.g., higher transcription/translation rate during exponential growth period, more ribosomes and more lingering rRNA in actively reproducing microbes). On the other hand, while 16S-RNA-seq presented minimal differentiations in spiked realistic samples, it did provide some insight into the discriminative ability between DNA and RNA libraries in samples of similar origin in our study (Fig. 3) as well as in a previous study with low biomass built environment samples [40]. These findings highlight the need for alternative approaches that accurately assess community activity in a way that allows us to further understand the basic biology of microbial communities and human-microbiome interactions.

The performance of 16S-RNA-seq in real microbial communities varied by environment, likely as a result of both biological and technical factors, including the biochemical characteristics of each sample, the biology of the microbes within communities (e.g., gene copies in the bacterial genomes, transcription rate, stability difference from microbe to microbe), and community composition (microbial diversity, viability, etc.). Previous reports have indicated that many biochemical factors can influence 16S-RNA-seq readouts [12]. Especially in non-host-associated contexts, moisture level, pH, light (UV) exposure, and the presence of nucleases can all vary dramatically among microenvironments, affecting the longevity of rRNA molecules as well as their absorption to their matrices (dust and other small particles) [46–48]. This biochemical phenomenon may help explain the non-differentiable results in soil and some of the BE samples (Figs. 2 and 4a), where highly moisturized (soil, bathroom), particle-rich (soil, outdoor/MBTA samples), and light (UV)-protected (bathroom, kitchen, and other indoor surfaces) environments tended to retain rRNA molecules longer, thus diminishing the differences between active vs. whole communities. The greater biochemical diversity and instability in such environments are usually also accompanied by greater microbial diversity and by more extensive differences in microbial activity, further affecting 16S-RNA-seq results. It should be noted that the physical and biochemical characteristics of BEs also bring additional technical challenges to accurate interpretation of 16S-RNA-seq readouts, inasmuch as individual protocol steps can also be differentially affected by different environmental conditions. Increasing this potential complexity, different microbial members of these communities may also be differentially affected — DNA/RNA yields may not be comparable directly after extraction, the efficiency of reverse transcription of RNA molecules can differ, and library construction may be variable among different environments. These technical factors also contribute to making 16S-RNA-seq somewhat more consistent in lower diversity, less biochemically complex environments, as also observed in this study [12, 38, 49].

Despite these potential limitations, ribosomal RNA has been widely used to investigate active microorganisms in human and environmental samples on the basis that they are relatively stable and accessible both inter- and intracellularly [12]. However, this raises the question of whether rRNA in microbial communities accurately represents which microorganisms are active in “real time.” The half-life of recently produced rRNA in soil bacterial communities was reported to have strong temperature dependency, which increased from days to over a year as the temperature decreased [50]. This suggests that rRNA may remain long after the microbe is dead and thus may not accurately indicate activity, especially when the samples are from chemically and geographically diverse backgrounds. Aside from the persistence of rRNA molecules, the 16S rRNA gene is not linearly correlated with actual bacterial count by nature [12, 51]. Copy number of the 16S rRNA gene varies greatly across microbial species. This does not even consider the gene’s transcription rate, which varies with factors such as growth rate, life stages, and exposure to stressors [12, 52]. Thus, 16S RNA/DNA abundance ratios vary between and within microbial communities. Particularly, some active, highly transcribable taxa may look “dormant” within a mixed community, as dormant cells may accumulate high numbers of ribosomes [1]. On the other hand, it is also possible that dead microorganisms or those with low metabolic output would appear as active, given the large amount and persistence of rRNA molecules [44]. This is worth considering especially in BE samples, where desiccation, regular disinfection, and lack of nutrients contributed to a relatively harsh environment and thus more microbes being dead or dormant [6]. The higher RNA/DNA ratios of the Lachnospiraceae and Tissierellaceae families in stool samples therefore do not necessarily indicate their being “more active” compared to BE or oil facilities (Fig. 4c) but a reiteration of the biological differences (i.e., life stages, microbial compositions) in the underlying microbial communities. Quantitation of active microbes using ribosomal RNA transcripts would thus be affected by the community structure as well as environments biochemical background — e.g., the readouts from 16S-RNA-seq would not linearly reflect the active composition outside very simple communities that contain mono- or closely related species and that are growing at a similar rate, which closely resembles our results (Fig. 1).

16S-RNA-seq was qualitatively and semiquantitatively informative in simple synthetic communities, but it is not always effective in complex communities and only indicates relative activity trends in BE samples. It is thus important to set proper criteria for better interpretation of 16S-RNA-seq results. This may be achieved by filtering the taxa based on appropriate prevalence or absolute abundance (read count) so that some systematic interference, such as contamination or taxa ubiquitous in those environments, may be minimized. This, however, risks losing information on rare taxa, which were reported being more differentially abundant in RNA vs. DNA libraries in BE samples [18, 40]. As in shotgun metagenomic sequencing, microbial community structure represented by 16S-RNA-seq is dependent on sampling effects and sequencing depth [7, 44, 53]. This is particularly true in activity assays in low biomass BE samples, where many rare taxa can only be detected in larger read libraries, and the differences between RNA and DNA libraries can be misrepresented due to the sampling stochasticity [12]. This explained some of the “phantom taxa” (taxa detected in RNA libraries by not the DNA libraries) [20] across various families (Fig. 4c) — the disproportionately high activity of those rare taxa is more likely a technical flaw than a real sign of activity. Therefore, it is important to consider the ceiling of sequencing technologies (depth, sampling statistics) when interpreting 16S-RNA-seq readouts in complex communities.

Another potentially helpful consideration is to include a threshold 16S abundance ratio to determine active taxa [54]. A ratio threshold of 0.1 to 10 might simply provide a conservative view of activity in microbial communities (Supplementary Fig. 6). Additionally, ensuring that the average abundances of a taxon in both RNA and DNA libraries are sufficient for confident detection can help ameliorate the effects of sequencing depth. For example, we can be fairly confident that the Comamonadaceae family is likely to be less active in indoor air samples given its high DNA/RNA abundance, which makes it less possible to be misrepresented by technical variance (sampling effect, sequencing depth, etc.). Similarly, the Tissierellaceae family, less active in oil facilities, is more transcriptionally active in stool samples and well-detected as such (Fig. 4c). However, for the reasons introduced above, it is not possible from sequencing alone to determine that these consistently enriched/depleted families simply have more/less persistent rRNA molecules within these particular environments. Overall, the results of 16S-RNA-seq may be better interpreted with the consideration of proper filtration, sequencing parameters, and overall abundance of the taxa.

A comprehensive evaluation of 16S-RNA-seq in microbial communities would require extensive effort, and there are limitations of the current study as a result. Our synthetically co-cultured and spiked “communities” use only a very small number of representative microbes, if anything leading us to underestimate the variability of 16S-RNA-seq between protocols and settings. While it may be impossible to directly interpret 16S-RNA-seq results for accurate viability quantifications, the thoughts here are promising in terms of qualitative assessment: determining which microbes are generally more active in a (or similar) environmental source. Other potential improvements might include the combination of other microbiological experiments, such as metabolic capacity measurements that directly capture the metabolism activities [55–57], biochemical colorimetry that based in the membrane integrity of viable cells [58], or to explore alternative activity markers from mRNA transcribed from protein-coding genes. A small number of protein coding genes have also been proposed for microbial community viability profiling in previous work, such *rpoB*, *gyrB*, and *cpn60* [59–61]. These housekeeping genes are potentially conserved enough to be detected and amplified using universal primers as with the 16S rRNA gene while similarly retaining variable regions used to discriminate microbes at high resolution. mRNAs from such functional genes have shorter half-life compared to ribosomal RNA and may thus better represent currently active members of a community. Additionally, they present in one copy (or conserved copies) in bacterial genomes and are transcribed stably but exclusively in the active growth phase of the cellular life cycle, so that their copies directly correlate with the active bacterial amounts. If their transcripts could be targeted as universally and reliably as those of the 16S rRNA gene, this combination of properties would make these genes of potential interest for viability assessment as well. Last but not least, activity assessment in microbial communities will benefit substantially from multiomic integration, e.g., combining 16S-RNA-seq with functional indicators such as metatranscriptomic or metaproteomic profiles. These somewhat circumvent the drawbacks of using 16S rRNA gene as activity markers, providing a complementary definition of viability, and directly observing activities such as virulence, pathogenicity, or antimicrobial resistance that are not captured by amplicon sequencing.

## Conclusions

In conclusion, our results found that while 16S-RNA-seq alone may never fully quantify viability in microbial communities, it can provide a qualitative profile of which community members are generally viable across similar environments and remain to be coupled with additional molecular approaches to understand the mechanisms of persistence, metabolism, and potential health consequences in the BE, environmental, and human microbiomes [62, 63].

## Supplementary Information

**Additional file 1:** **Supplementary File 1.** Metadata of this study.

**Additional file 2:** **Supplementary File 2.** OTU table obtained from bioBakery workflow of this study.

**Additional file 3:** **Supplementary File 3.** Statistical tests results. 

**Additional file 4:** **Supplementary File 4.** Metadata of five studies.

**Additional file 5:** **Supplementary File 5.** OTU table obtained from bioBakery workflow of five 16S-RNA-seq studies.

**Additional file 6:** **Supplementary File 6.** Differentially significant taxa in the comparative study (part 4) from MaAsLin 2

**Additional file 7:** **Supplementary Figure 1.** Approval from the MBTA. We received the letter of approval from MBTA, by way of the General Manager’s Office, to carry out the study and confirmed the detailed sampling plans with the MBTA prior to any public work. Their assistance and input were invaluable both for study design and for safe execution of sample collection, and the letter includes the initial information from Evan Rowe approving the work.

**Additional file 8:** **Supplementary Figure 2.** Taxonomic composition of control samples. Relative abundance of eight control samples is presented here, prepared from swabs dipped (exposed) in 10^2^ *E.coli* culture, 10^6^* E.coli* culture, indoor air, outdoor air, blank culture broth, one blank DNA extraction buffer (EB buffer), one distilled water and one sterile 0.85% saline used in the lab space.

**Additional file 9:** **Supplementary Figure 3.** Determining microbial biomass of realistic community samples with(out) spike-in (part 2). qPCR was performed targeting 16S rRNA gene V4 region in DNA and RNA (cDNA) extractions from samples collected from four computer screens, four computer mice, three human saliva and three soil. Each subject was sampled in parallel, with one set spiked with 10^2^CFU (computer screens and computer mice) or 10^7^ CFU (saliva and soil) *E. coli *culture.

**Additional file 10:** **Supplementary Figure 4.** Relative abundance of six differentially abundant taxa in DNA and RNA libraries in subway samples (part 3). Each column represents the average relative abundance of respected taxa in four DNA (labelled as sample(DNA)) or RNA (sample(RNA)) libraries. Dot represents each sample. FDR adjusted *q* values were calculated from mixed effects linear model (MaAsLin2), listed in Supplementary File 6.

**Additional file 11:** **Supplementary Figure 5.** Relative abundance of the most abundant taxa in different samples across different sample types. Relative abundance of the 15 most abundant taxa in samples of oil facilities, built environment (bathroom, kitchen surfaces, computer screens and mice, subway seats, subway walls, subway grips and touchscreens of ticket machines and indoor air), human saliva, soil, human stools.

**Additional file 12:** **Supplementary Figure 6.** Heatmap presenting 16S RNA/DNA ratios of six differentially abundant families in different samples (part 4). Log10 RNA vs. DNA Relative abundance ratio was calculated for each genus within each family.

## Data Availability

The sequencing data of this article are available in the NCBI SRA repository, under BioProject number: PRJNA859497. The scripts used in this study are available at the GitHub repository: https://github.com/biobakery/Viability_16S-RNA-seq_Microbiome.
