# Supplementary figures and images for: RNA-based amplicon sequencing is ineffective in measuring metabolic activity in environmental microbial communities
Source: Microbiome. 2023 Jun 13;11:131. doi: 10.1186/s40168-022-01449-y (PMC10262425; doi:10.1186/s40168-022-01449-y)

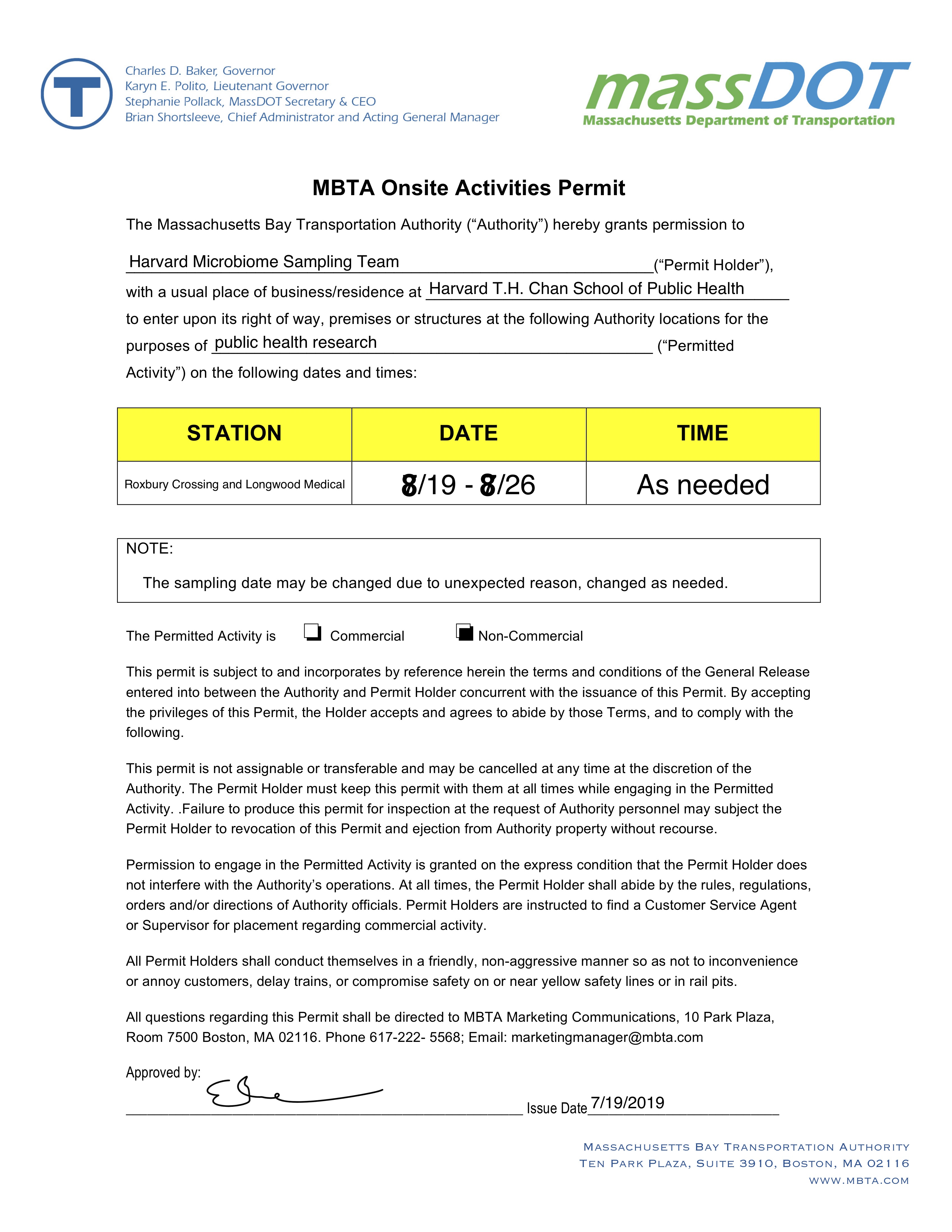

Supplement: Supplementary file 8 — Additional file 7: Supplementary Figure 1. Approval from the MBTA. We received the letter of approval from MBTA, by way of the General Manager’s Office, to carry out the study and confirmed the detailed sampling plans with the MBTA prior to any public work. Their assistance and input were invaluable both for study design and for safe execution of sample collection, and the letter includes the initial information from Evan Rowe approving the work. [file 40168_2022_1449_MOESM7_ESM.png]

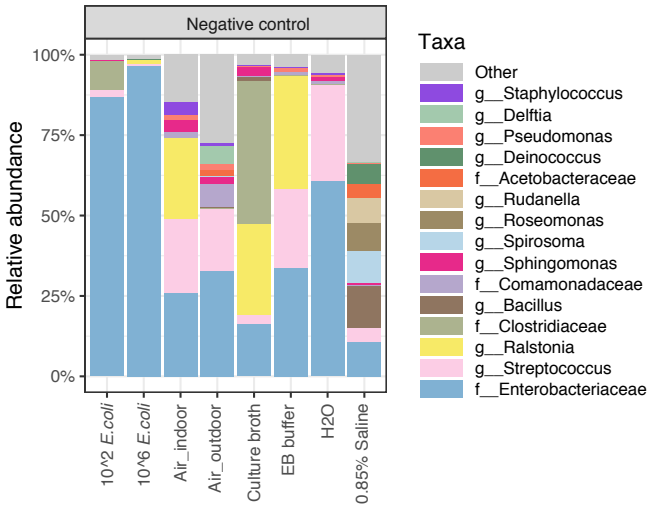

Supplement: Supplementary file 9 — Additional file 8: Supplementary Figure 2. Taxonomic composition of control samples. Relative abundance of eight control samples is presented here, prepared from swabs dipped (exposed) in 102 E.coli culture, 106 E.coli culture, indoor air, outdoor air, blank culture broth, one blank DNA extraction buffer (EB buffer), one distilled water and one sterile 0.85% saline used in the lab space. [file 40168_2022_1449_MOESM8_ESM.pdf]

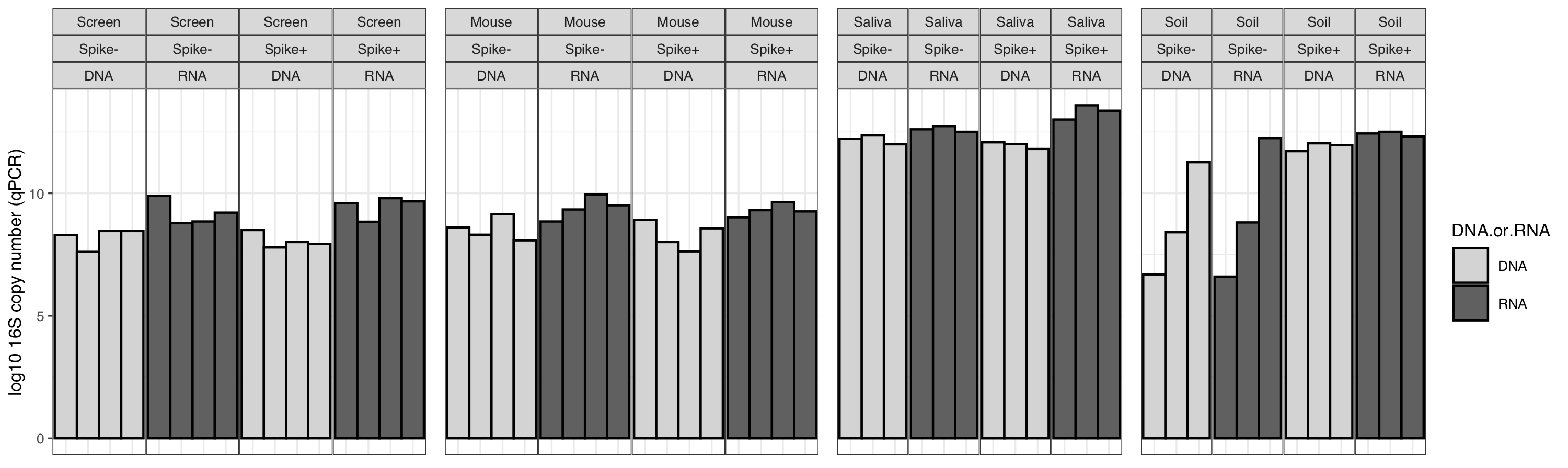

Supplement: Supplementary file 10 — Additional file 9: Supplementary Figure 3. Determining microbial biomass of realistic community samples with(out) spike-in (part 2). qPCR was performed targeting 16S rRNA gene V4 region in DNA and RNA (cDNA) extractions from samples collected from four computer screens, four computer mice, three human saliva and three soil. Each subject was sampled in parallel, with one set spiked with 102CFU (computer screens and computer mice) or 107 CFU (saliva and soil) E. coli culture. [file 40168_2022_1449_MOESM9_ESM.png]

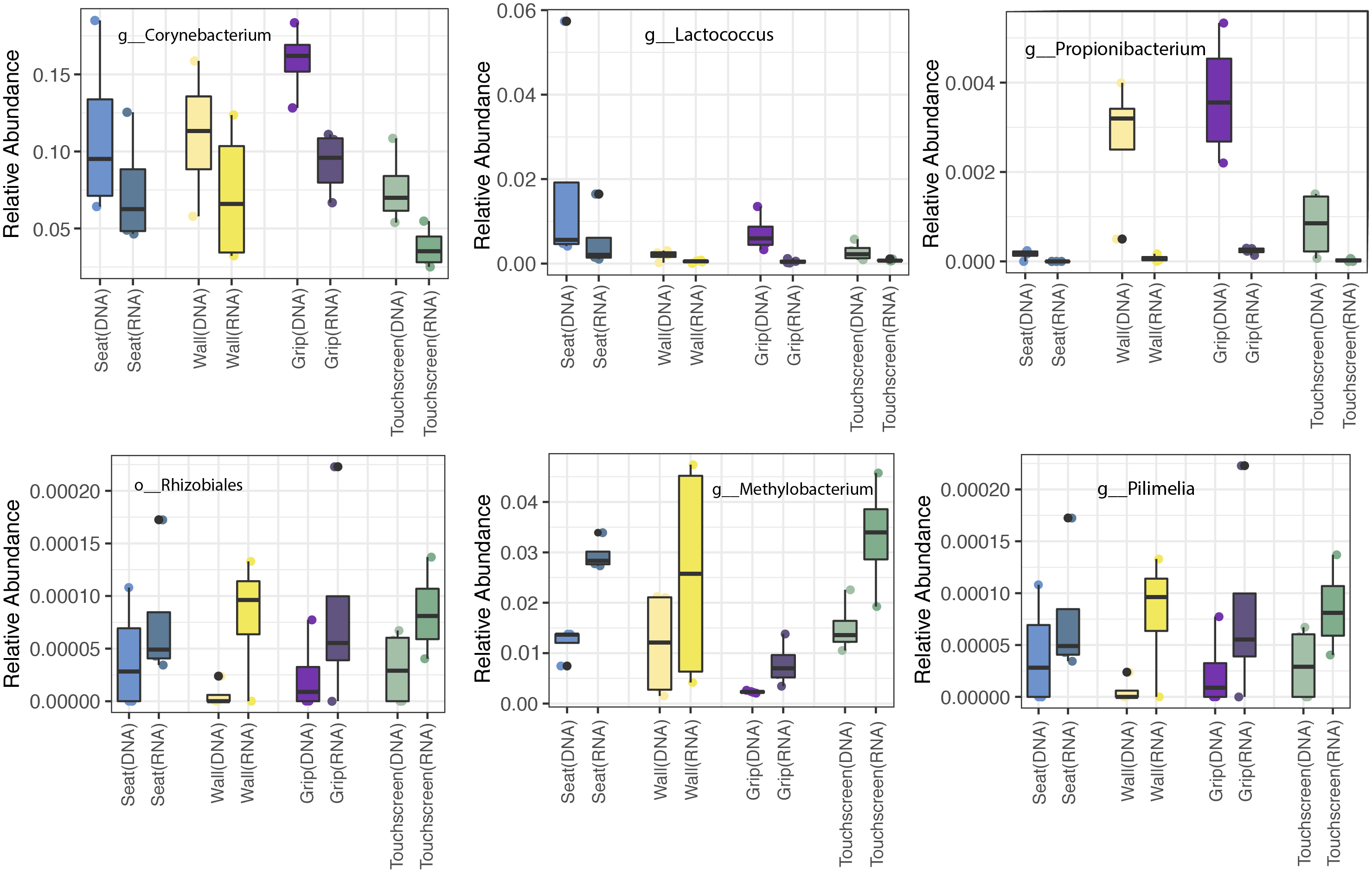

Supplement: Supplementary file 11 — Additional file 10: Supplementary Figure 4. Relative abundance of six differentially abundant taxa in DNA and RNA libraries in subway samples (part 3). Each column represents the average relative abundance of respected taxa in four DNA (labelled as sample(DNA)) or RNA (sample(RNA)) libraries. Dot represents each sample. FDR adjusted q values were calculated from mixed effects linear model (MaAsLin2), listed in Supplementary File 6. [file 40168_2022_1449_MOESM10_ESM.png]

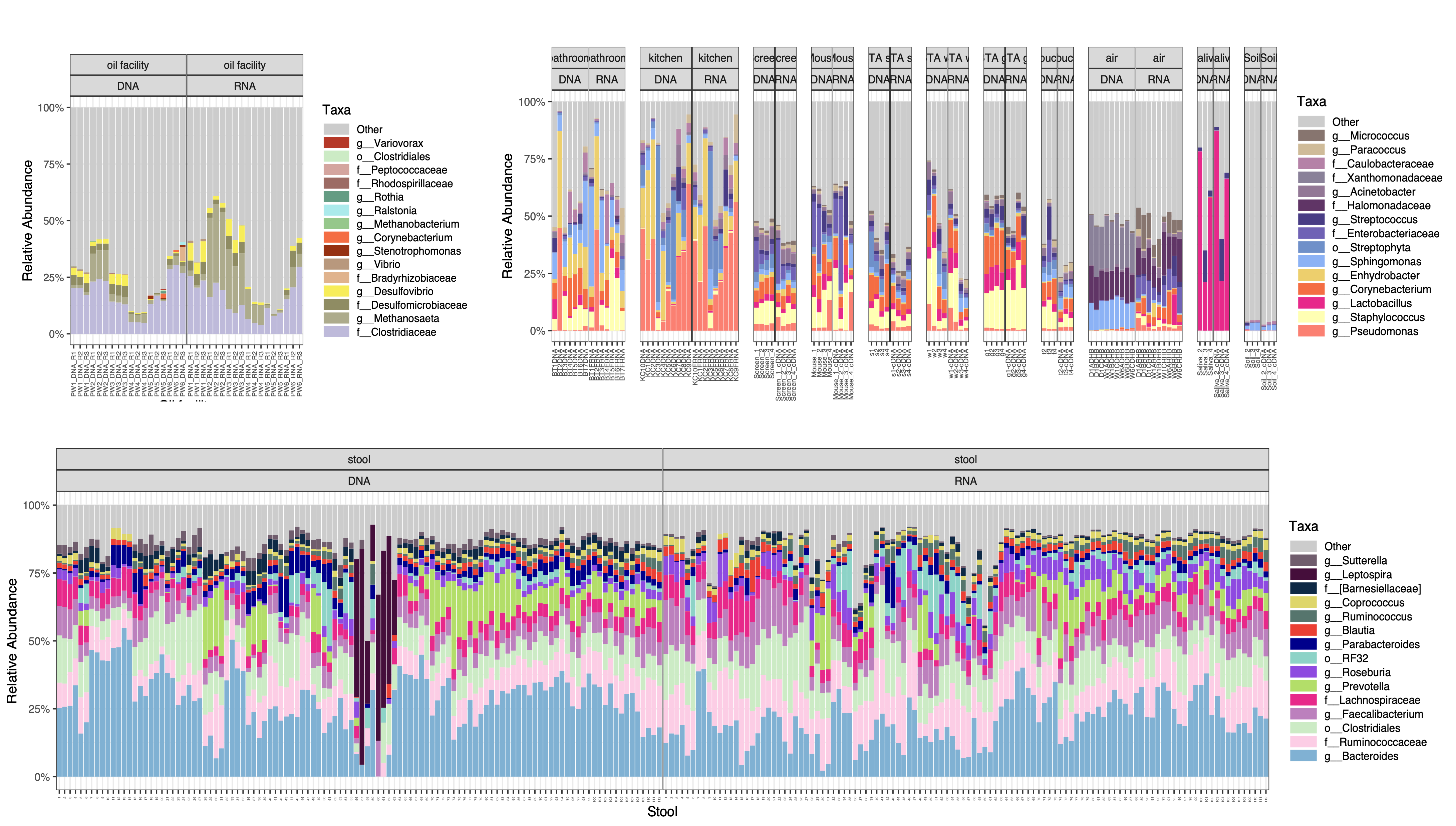

Supplement: Supplementary file 12 — Additional file 11: Supplementary Figure 5. Relative abundance of the most abundant taxa in different samples across different sample types. Relative abundance of the 15 most abundant taxa in samples of oil facilities, built environment (bathroom, kitchen surfaces, computer screens and mice, subway seats, subway walls, subway grips and touchscreens of ticket machines and indoor air), human saliva, soil, human stools. [file 40168_2022_1449_MOESM11_ESM.png]

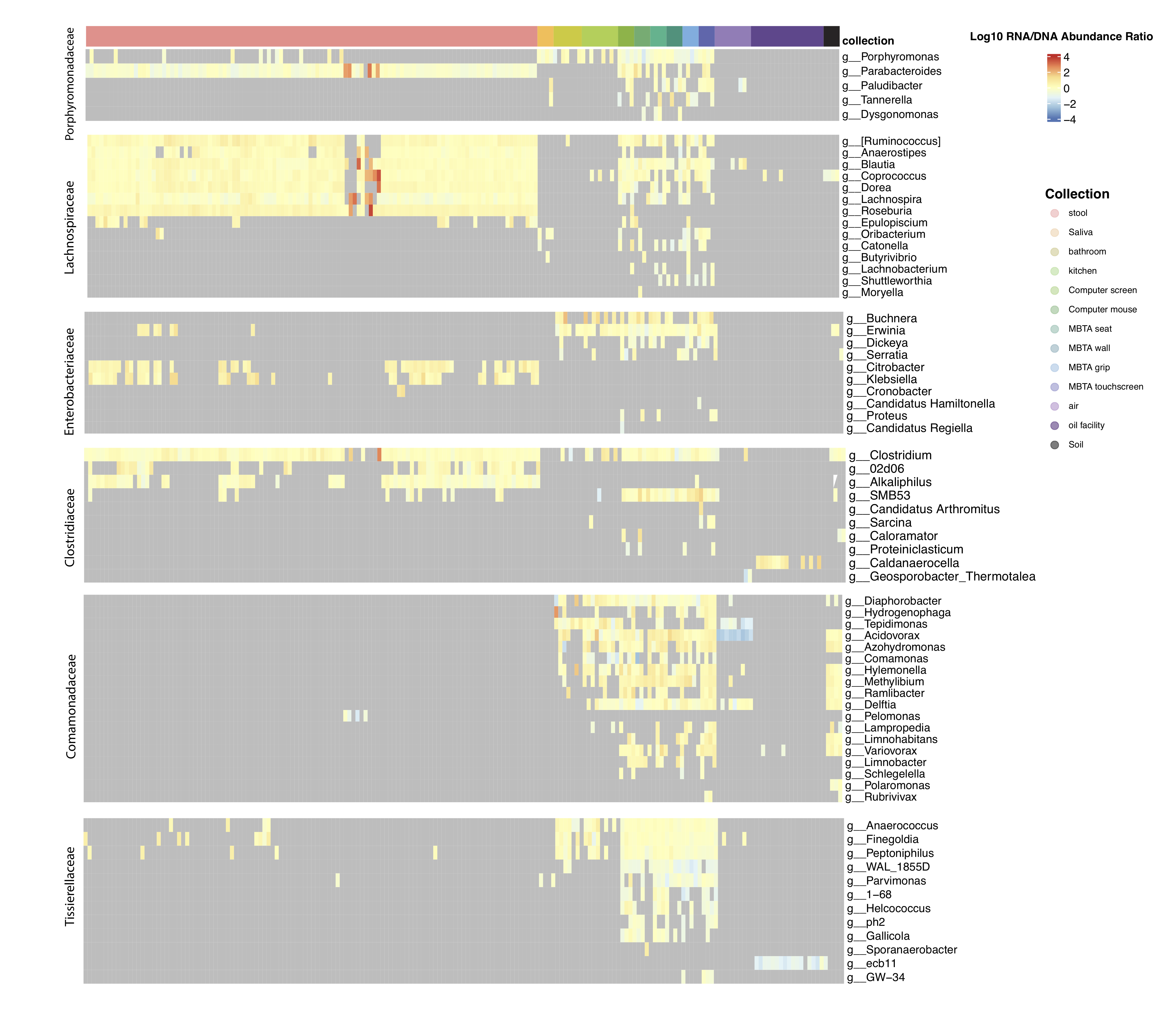

Supplement: Supplementary file 13 — Additional file 12: Supplementary Figure 6. Heatmap presenting 16S RNA/DNA ratios of six differentially abundant families in different samples (part 4). Log10 RNA vs. DNA Relative abundance ratio was calculated for each genus within each family. [file 40168_2022_1449_MOESM12_ESM.png]
